# Supplementary material for: The emergence of three-dimensional chiral domain walls in polar vortices
Source: Nat Commun. 2023 Jul 25;14:4465. doi: 10.1038/s41467-023-40009-2 (PMC10368707; doi:10.1038/s41467-023-40009-2)
Supplement: Supplementary file 1 — Supplementary Information [file 41467_2023_40009_MOESM1_ESM.docx]

Supplementary Text for:

The emergence of three-dimensional chiral domain walls in polar vortices

Sandhya Susarla^1,2,$#*^, Shanglin Hsu^1,2#^, Fernando Gómez-Ortiz^3^, Pablo García-Fernández^3^, Benjamin H. Savitzky^1^, Sujit Das^4^, Piush Behera^5^, Javier Junquera^3^, Peter Ercius^1^, Ramamoorthy Ramesh^1,2,5,6,7,8*^, Colin Ophus^1*^

1: National Center for Electron Microscopy, Molecular Foundry, Lawrence Berkeley National Laboratory, Berkeley, CA, USA 94720

2: Materials Sciences Division, Lawrence Berkeley Laboratory, Berkeley CA, USA 94720

3: Departmento de Ciencias de la Tierra y Física de la Materia Condensada, Universidad de Cantabria, Cantabria Campus Internacional Santander, Spain, 39005

4: Materials Research Centre, Indian Institute of Science, Bangalore, 560012, Karnataka, India

5: Department of Materials Science & Engineering, University of California, Berkeley, CA, USA 94720

6: Department of Physics, University of California, Berkeley

Berkeley, CA, USA 94720

7: Department of Physics, Rice University, Houston, TX, USA, 77005

8: Department of Materials Science and Nanoengineering, Houston, TX, USA, 77005

#Equal contribution.

$ Present address: School for Engineering of Matter, Transport, and Energy, Arizona State University, Tempe, 85280, AZ, USA

Corresponding authors: sandhya.susarla@asu.edu, [ramamoorthy.ramesh@rice.edu](mailto:ramamoorthy.ramesh@rice.edu), and cophus@gmail.com

**Figure S1:** Polarization vector maps overlaid on the drift-corrected HAADF-STEM images. The yellow vector indicates the direction of polarization. The underlying red/blue contrast is the curl of the displacement.


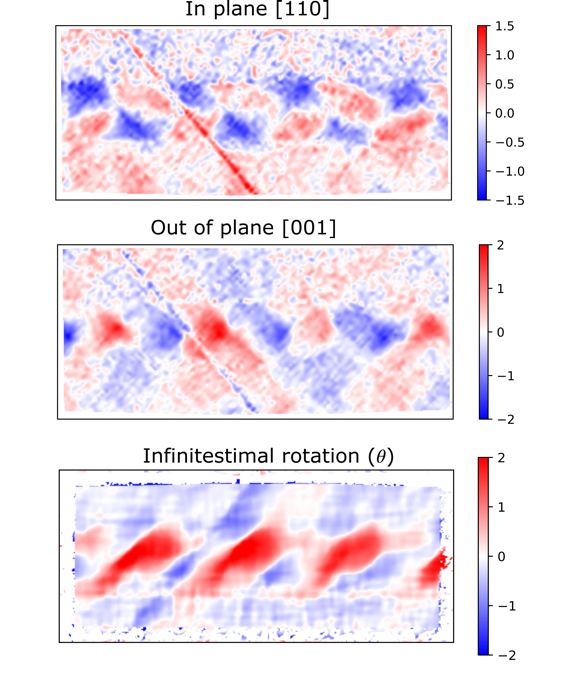


**Figure S2:** Strain maps (top two) and infinitesimal rotation (bottom) extracted by A-site fitting of atoms in Figure S1.

**Line profile:** The line profiles from the images were extracted from across the different chiral grain boundaries. In conjunction in with polarization maps in Figure 2, we observe that the axial and lateral polarization are antiparallel across 𝛼 and 𝛽 domain boundaries respectively. On the other hand, both axial and lateral polarization are antiparallel across the 𝛾 domain boundaries.

**Mathematical Representation of Chirality:** Assuming that we measure the axial and lateral components of the polarization at the topmost PbTiO_3_ (PTO) layer Figure S5 (a) and assuming a vortex geometry where the polarization lines are closed a vortex is univocally determined in Figure S5 (b). The polarization vector field defined by this vortex in the (x,z) plane is $\vec{p_{x,z}}=p_{lateral(z,-x)}$ whose curl takes the value of directed along the axial direction. Therefore, helicity Equation 1 can be approximated to $\mathcal{H=}2\cdot<p_{lateral}>\cdot<p_{axial}>$

**Statistics**: We also repeated the 4D STEM analysis on the other datasets and the results are shown in Figure S6-S7.

**Figure S3:** Line profiles showing the difference lateral and axial polarization in the 𝛼, 𝛽 and 𝛾 domain boundaries.

**Figure S4:** Dark field images corresponding disks 1-4.

**
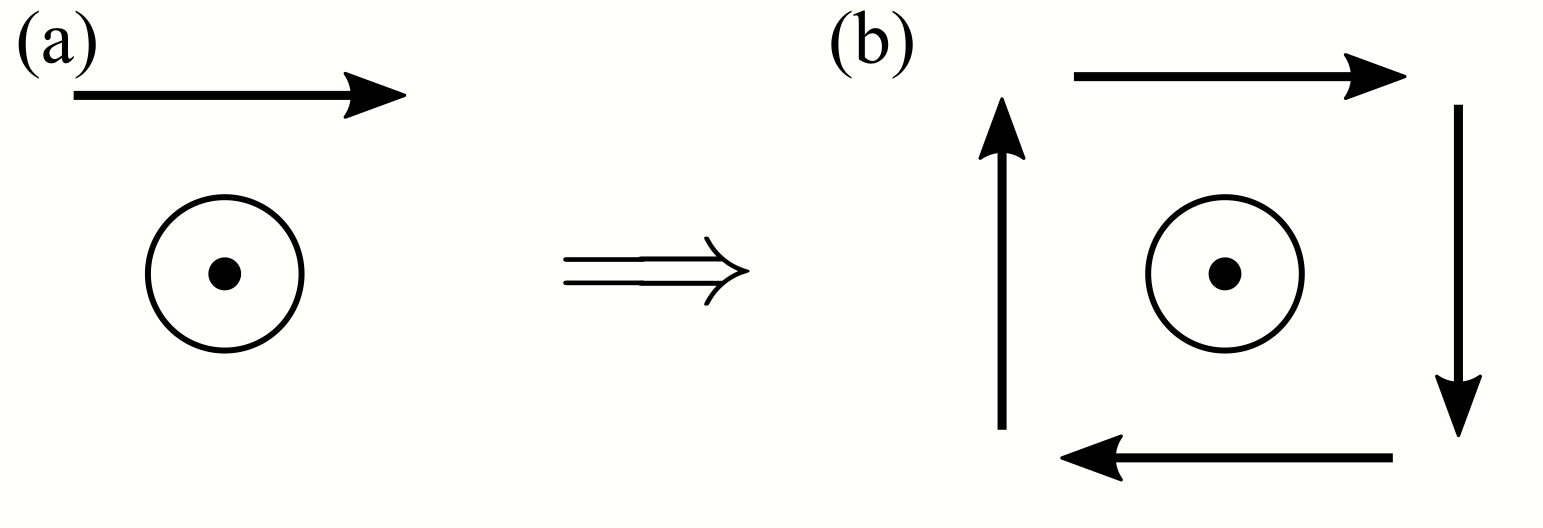
**

**Figure S5:** Schematic representation of the vortex topology. (a) Lateral and axial components were measured at the topmost layers of PTO. (b) Geometry obtained after assuming a vortex structure from the polarization texture of (a)

**Figure S6:** Virtual image, polarization, and helicity maps from different 4D STEM datasets showing the repeatability of different chiral/achiral boundaries in the PTO/STO trilayer. The presence of triple point topologies is evident whenever the chiral and achiral boundaries intersect one another. Scale bar: 20 nm for all panels.

**Figure S7:** Virtual image, polarization, and helicity maps from different 4D STEM datasets showing the repeatability of different chiral/achiral boundaries in the PTO/STO trilayer. The presence of triple point topologies is evident whenever the chiral and achiral boundaries intersect one another. Scale bar: 30 nm
